# Immunomodulatory Effects of Copper Bis-Glycinate In Vitro

**DOI:** 10.3390/molecules30061282

**Published:** 2025-03-13

**Authors:** Alexander Areesanan, Luise Wolf, Sven Nicolay, Amy Marisa Zimmermann-Klemd, Carsten Gründemann

**Affiliations:** Translational Complementary Medicine, Department of Pharmaceutical Sciences, University of Basel, Campus Rosental—Mattenstrasse 22, CH-4058 Basel, Switzerland; alexander.areesanan@unibas.ch (A.A.); l.wolf@unibas.ch (L.W.); sven.nicolay@unibas.ch (S.N.); amy.klemd@unibas.ch (A.M.Z.-K.)

**Keywords:** copper bis-glycinate, food supplement, inflammation, antioxidant, immunomodulatory effects

## Abstract

Copper functions as a cofactor and antioxidants in a large number of enzymes that are important for cellular respiration and the nervous system. In the last century scholars have explored copper’s relationship with the immune system, with copper deficiency drastically upsetting the overall function of the immune system, as seen in symptoms such as increased susceptibility to pathogens, decreased proliferation of lymphocytes, and impaired function of both cytotoxic T lymphocytes and helper T cells. Among copper’s various forms, copper bis-glycinate (Cbg) has been used as an official EU-approved oral supplement to promote health. In this study, we observed the influence of Cbg on human epithelial cells (HCE-T cells) to determine its cytotoxicity, anti-reactive oxygen (ROS), and wound healing capabilities. We also evaluated Cbg’s anti-inflammatory immune cells like primary human mononuclear cells (PBMCs), monocytic THP-1, and Jurkat cells in the context of anti-inflammation. At all the investigated concentrations of Cbg (0.05–100 μg/mL), ther was no considerable impact detected on the epithelial cells. However, the proliferation rate of stimulated PBMCs was affected progressively (3–50 μg/mL). In CD4^+^ helper T cells, interleukin (IL)-17 and IL-2 cytokine levels were decreased in a dose-dependent manner, while interferon (IFN)-γ and IL-2 levels were slightly decreased with no noticeable changes between each treated concentration. Furthermore, stimulated monocytic THP-1 cells treated with Cbg reduced IL-6 and significantly reduced tumor necrosis factor (TNF)-α cytokines secretion. Lastly, stimulated Jurkat intracellular Ca^2+^ influx was significantly inhibited in a dose-dependent manner. Taken together, this study demonstrated that copper possesses modulatory effects on immune cells but not on epithelial cells, but further studies are needed to underline this hypothesis.

## 1. Introduction

The human immune system fulfils diverse and complex functions. Inflammatory reactions initiated by the immune system serve to protect against infections caused by invading pathogens. The immune system also recognizes tissue damage and triggers repair mechanisms. If this leads to persistent or excessive inflammation—possibly due to a dysfunction of the complex coordinated reactions—autoimmune diseases such as rheumatoid arthritis or multiple sclerosis can develop [1,2]. Various classes of immunosuppressants are generally used in the treatment of autoimmune diseases [3]. Glucocorticoids, as well as cell cycle inhibitors (cyclophosphamide or mycophenolate), are effective, but this effectiveness is linked to a number of side effects [2,4,5]. Small molecule drugs (e.g., cyclosporine A, tacrolimus, or tofacitinib) that interfere with T cell signaling, or the far more expensive highly specific biologics, are also frequently used today. However, the strong intervention in the immune system increases susceptibility to infection and can cause paradoxical inflammation [3,5,6,7].

It is recommended that humans need 0.9 mg of copper per day, which is absorbed in the small intestine and then delivered to other organs to form apo-enzymes [8]. The blood level of copper is physiologically between 11 and 25 μmol/L [9]. Copper is known for its functions as a catalytic cofactor of more than 300 proteins in mammals, such as methionine, histidine, and cysteine. Due to its oxidative properties, the human organism utilizes copper as a cofactor in numerous enzymatic functions, such as mitochondrial respiration (cytochrome oxidase [COX]), collagen cross-linking lysine oxidase [LOX]), and antioxidation (superoxide dismutase [SOD]) [10]. For instance, the mineral is essential for various processes in the body, such as brain function and the formation of connective tissues and blood vessels, as well as energy metabolism [11]. It is also known to be important for the functioning of the immune system [11]. Consequently, a lack of copper can lead to an inadequate immune response; for example, through a reduced number of neutrophils [12] or a reduced production of superoxide anions [13]. Furthermore, copper deficiency reduces the function of the adaptive immune system (e.g., a reduced number of antibody-producing B cells [14] and interleukin [IL]-2 secretion by helper T cells [15,16]). Infection increases serum copper concentrations and interleukin (IL)-1-mediated synthesis of the protein ceruloplasmin in the liver, which stores and transports copper [17].

In addition to the importance of copper for a functioning immune system, the role of the mineral in excessive immune reactions (e.g., rheumatoid arthritis) has also been investigated and discussed. As early as 1978, Whitehouse and colleagues found a moderate anti-inflammatory and anti-arthritic effect of various copper salts in rats [18]. A number of older studies have subsequently demonstrated the anti-inflammatory effects of copper in various in vitro and in vivo models [19,20,21,22]. One such study showed that copper sulphate suppresses the secretion of the cytokines IL-1β and IL-6 by human peripheral blood leukocytes [23]. In 2006, the toxicity of copper on isolated human peripheral blood mononuclear cells was investigated. The results show a clear suppression of proliferation in the thymidine uptake assay, based on cytotoxic effects (LD_50_: 115 µM) [24]. A study from 2016 investigated both the cytotoxicity and the proliferation rate, as well as the influence of copper nitrate on cytokine secretion (IL-2 and IFN-γ secretion) in primary human mononuclear cells, revealing inhibitory effects at sub-toxic concentrations [9]. More recently, researchers have investigated a possible influence of copper-infused fabric on the function of human macrophages. The authors showed that copper could block the secretion of inflammatory cytokines by LPS-treated macrophages. In addition, copper significantly reduced NF-κB and IRF3 activation in LPS-stimulated macrophages [22].

Allergic rhinitis (AR) is part of a systemic allergic reaction characterized mainly by nasal symptoms (e.g., nasal congestion, sneezing, and clear nasal discharge) [25]. However, other symptoms such as conjunctivitis, non-productive cough, or sinusitis may also occur [25]. Inhalation of allergens causes an immunoglobulin E (IgE)-mediated reaction of type 2 helper cells (Th2) [25]. A massive histamine release by mast cells then causes the symptoms described. Leukotrienes and prostaglandins affect the blood vessels, causing the characteristic nasal congestion [25]. The correlation between serum copper levels and AR occurrence was previously examined in a clinical study in adults in 2011 [26]. The study showed that the patient group with AR (n = 22) had, on average, a 32% higher serum copper level (0.79 µg/mL vs. 0.59 µg/mL, *p* < 0.001) compared to the control group (n = 36) [26]. A recent study in 2022 confirmed this trend, finding slightly elevated copper levels in patients with AR (n = 35) compared to healthy controls (0.111 vs. 0.104 µg/mL) [27]. However, these were not significant (*p* = 0.328) [27]. Moreover, a study published in 2020 examined the question of whether copper levels increase in AR pathology. The study found that 7–9-year-old children with AR (n = 31) already had been supplied with significantly (*p* = 0.048) higher levels of copper (0.66 µg/mL) in their cord blood compared to the total cohort (n = 211, 0.47 µg/mL) [28]. Higher copper content in cord blood thus seems to favour the occurrence of AR. However, another 2022 study came to the interesting conclusion that dietary copper intake is negatively associated with the occurrence of AR [29]. The study participants with AR (n = 186) had an average intake of 1.41 µg/10 mJ copper, whereas the control group (n = 106) had 1.48 µg/mJ [29].

Overall, copper’s bifunctional (stimulating and inhibiting) effects on the immune system and in the development of allergic and excessive immune reactions have undergone extensive research. However, data on the anti-inflammatory effects of copper are currently rather sparse. Nonetheless, the results that have been published suggest that a more in-depth investigation could be worthwhile. There are currently several copper bis-glycinate (Cbg, C_4_H_10_CuN_2_O_5_) preparations on the market. Cbg is a chelated form of copper that is better absorbed by the body than other mineral sources (e.g., copper sulfate) [30], as it binds to the amino acid glycine, forming a chelated compound. The glycine molecule helps copper be absorbed more easily, resulting in a higher absorption rate compared to other forms of copper [30].

This study investigated and compared the in vitro anti-inflammatory potential of Cbg on human epithelial (HCE-T) cells versus immune cells such as primary human mononuclear cells (PBMCs), monocytic THP-1, and Jurkat cells.

## 2. Results

### 2.1. Free Radical Scavenging Capacity of Cbg

**Figure 1:**
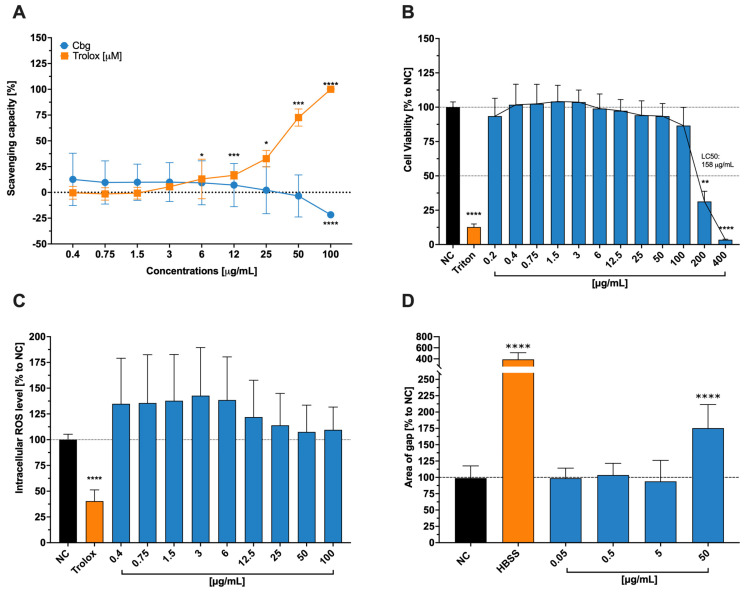
Influence of CbG (blue) on free-radical scavenging capacity (**A**), cell viability (**B**), intracellular ROS levels (**C**), and wound healing (**D**) of epithelial cells (HCE-T). (**A**) Scavenging power was analysed (n = 4) and is shown with ± standard deviation. * *p*  <  0.05, *** *p*  <  0.001, **** *p*  <  0.0001. (**B**) Cells were incubated with medium (NC), Triton 100-X, or copper and are presented in comparison to NC as mean ± standard deviation (n = 4). ** *p*  <  0.01, **** *p*  <  0.0001. (**C**) Cells were incubated with H_2_DCFDA (25 µM) and copper and were then introduced to the UV light. Presented were the fluorescent intensity, normalized to the stimulated (UV-Light) cells (NC) and as mean ± standard deviation (n = 4). Trolox was used as a positive control. (**D**) After scratching, the cells were incubated with copper for at least 17 and imaged with a digital camera. Illustrated is the wound size normalized to control (NC) and presented as mean ± standard deviation (n = 4). **** *p*  <  0.0001.

Quantification of the Cbg’s antioxidant property is based on the ability to scavenge free radicals with a colorimetric reagent, which allows measurement by spectrophotometry (Figure 1A). This assay was performed due to the fact that high levels of radicals can elevate the immune response and induce inflammation, hence the importance of antioxidants like copper. The scavenging activities are determined by the ability of electron donors. Overall, different concentrations of Cbg showed no scavenging activity, in contrast to Trolox, where a significant dose-dependent manner was observed.

### 2.2. Effects of Cbg on Viability, ROS, and Wound Healing on Human Epithelial Cells

The viability of the cells was examined by determining their metabolic activities using a colorimetric reagent (Figure 1B). The described assays were used to represent the impact of Cbg on epithelial cells, which are part of the primary defense against foreign particles, microorganisms, etc., and they also possess important reactants of the innate immune system. Noticeable impacts on the viability of epithelial cells were detected at 100 μg/mL and above. The LC_50_ was determined at a concentration of 158 μg/mL, whereby strong cytotoxic effects were exhibited at 200 and 400 μg/mL. Moreover, to quantify the anti-ROS property in Cbg-treated HCE-T, a cell-permeated H_2_HDCFDA probe was used to quantify the intracellular ROS levels (Figure 1C). At all of the tested concentrations of Cbg, treated HCE-T displayed neither roles in stimulating antioxidant enzymes nor eliminating ROS, such as hydrogen peroxide and hydroxyl radicals. Furthermore, the gap area of the scratched confluence HCE-T cells was used to identify the impact of the Cbg on healing the wound (Figure 1D). No significant impacts were seen in regards to wound repair at the concentration between 0.05 and 5 μg/mL compared to the negative control. Conversely, a significant hindrance in the closure of the wound by 1.5-fold compared to the negative control was detected when treated at 50 μg/mL.

### 2.3. Effects of Cbg on THP-1 Viability and NF-κB Expression

**Figure 2:**
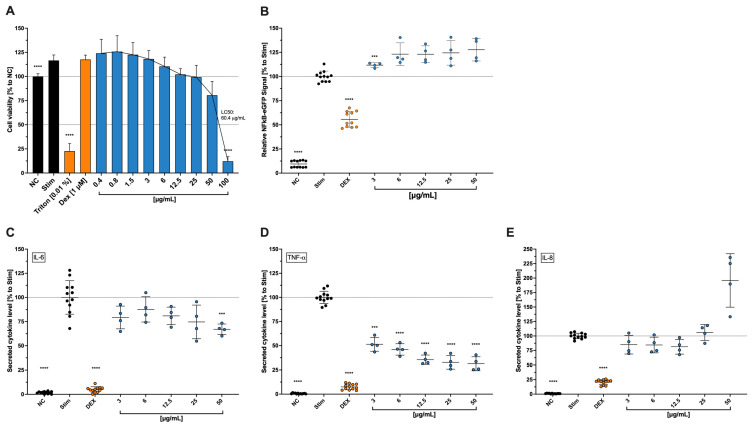
Influence of Cbg (blue) on the THP1-NF-κB viability (**A**), NF-κB expression (**B**), cytokine secretion of IL-6 (**C**), TNF-α (**D**), and IL-8 (**E**). Retrovirally transduced NF-κB-eGFP THP-1 human monocytic cells were stimulated with LPS (1 µg/mL) and treated for 24 h with extracts. The cell viability (**A**) was investigated by WST-1 and NF-κB expression was analysed by flow cytometry (**B**). The relative viable cells and NF-κB expression were normalized to the control (NC. and stim., respectively) and presented as mean ± standard deviation. n = 4. After the cells were stimulated and treated with Cbg for 6 h, the supernatants were collected and analysed with LEGENDplex™ to quantify the secreted cytokines (**C**–**E**). The relative cytokine concentration was normalized to the control (stim) and presented as mean ± standard deviation. n = 4; *** *p* < 0.001, **** *p*  <  0.0001.

In contrast to HCE-T viabilily, LC_50_ of treated THP-1 cells stimulated with LPS was determined at 55.3 μg/mL (Figure 2A). THP-1 cells’ viability decreased by approximately 20% at 50 μg/mL and by over 80% at 100 μg/mL. Furthermore, a slight increase in metabolic activities was observed in LPS-exposed cells without or with Cbg at low concentrations (0.4–6 μg/mL). Moreover, NF-κB, a transcription factor that activates inflammatory target genes, was also evaluated (Figure 2B). Cbg was shown to upregulate NF-κB expression in all tested concentrations (3–50 μg/mL). However, no dose-dependent manner was observed with approximately 25% increased expression compared to the untreated-stimulated cells.

### 2.4. Effects of Cbg on THP-1 Cytokine Secretion

The influence of Cbg on the secretion of pro-inflammatory cytokines by THP-1 cells was assessed (Figure 2C–E). Cbg slightly reduced IL-6 levels in the supernatants of LPS-exposed THP-1 at 3–25 μg/mL with a significant effect at 50 μg/mL, which reduced IL-6 levels by approximately 30%. Furthermore, significant inhibitory effects on TNF-α were identified when treated with Cbg. At concentrations of 3 μg/mL and above, over 50% of TNF-α levels were reduced compared to the untreated-stimulated cells. Nevertheless, its peak of 70% reduction of the cytokine release was measured at 25 μg/mL with no substantial changes at 50 μg/mL. In terms of IL-8 levels, minor decreased levels of approximately 20% were observed at 3–12.5 μg/mL, but not at 25 μg/mL. Although not significant, a higher level of increased IL-8 by almost 2-fold was detected at 50 μg/mL.

### 2.5. Effects of Cbg on Ca^2+^ Influx with Jurkat Cells and PBMC Stained with CFSE

**Figure 3:**
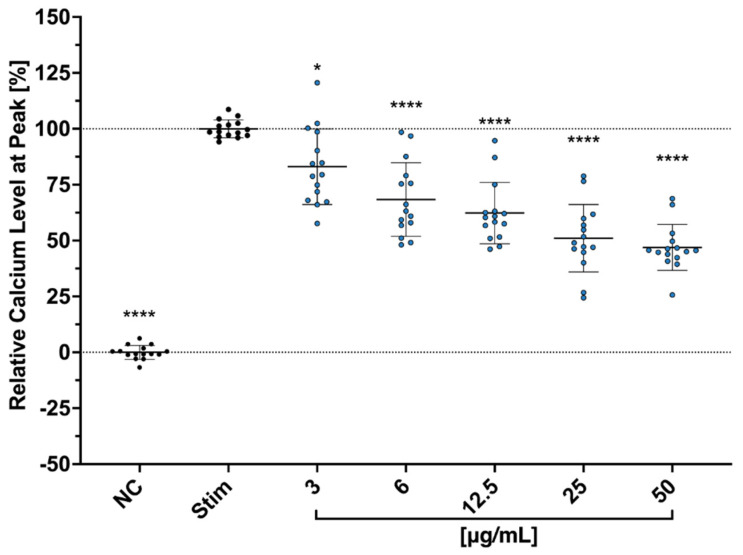
Impact of Cbg (blue) on Ca^2+^ influx. Jurkat cells were incubated with (Fluo3/AM) before exposing to Cbg and stimulated with CD3/CD28 antibodies. Flow cytometry was used to analyze the intracytoplasmic calcium level. The relative inhibition of calcium influx was normalized to the control (stim.) and presented as mean ± standard deviation. n = 5; * *p* <0.05, **** *p*  <  0.0001.

**Figure 4:**
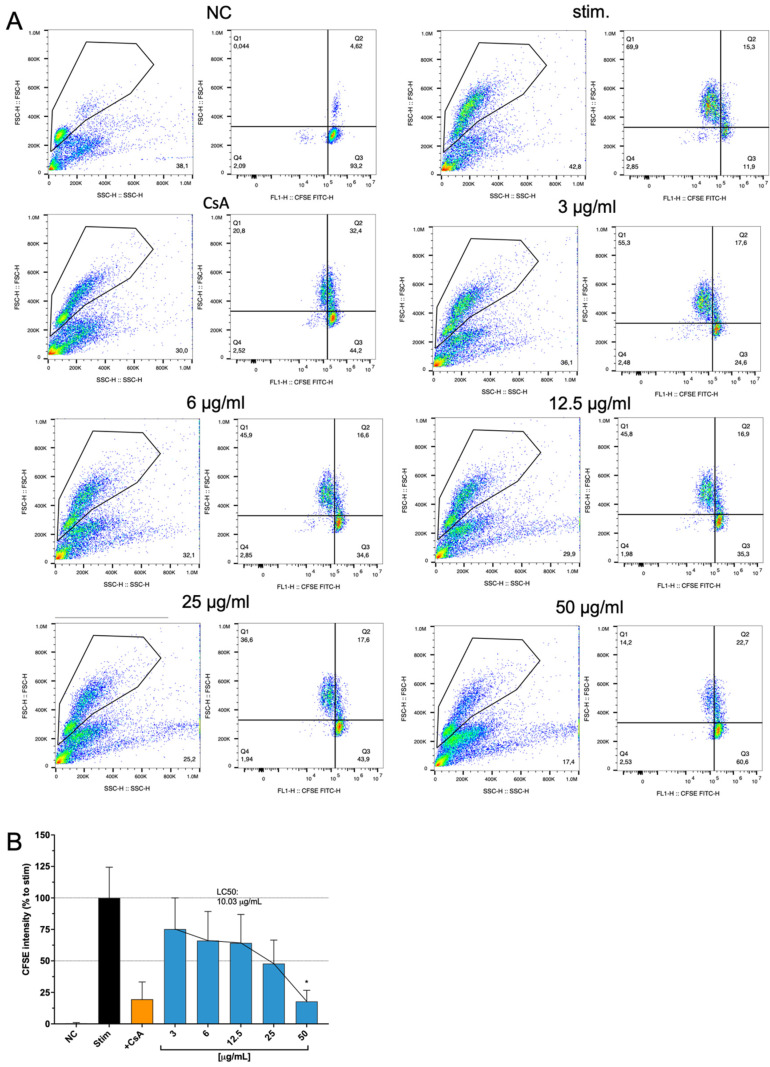
Impact of Cbg (blue in bar graph) on the proliferation of stimulated PBMCs. Stimulated cells were incubated for 72 h in the presence of medium only, CsA (5 μg/mL), or Cbg (3–50 μg/mL). The division of the CFSE-stained cells was then analysed by flow cytometry. Presented were single-concentration values (**A**) and the summary as bar graph (**B**). Results were normalized to positive control of untreated-stimulated cells and depicted as mean  ±  standard deviation. n  =  3; * *p*  <  0.05. Single concentration values were obtained by gating the CFSE signal (black circles). The colors in A illustrate where the highest concentrations of cells were found (blue = low concentration, red high concentration).

The influence of Cbg on the calcium flux of Jurkart cells was investigated and showed a clear dose-dependent effect (Figure 3). Proliferative function using CFSE combined with flow cytometry was tested on Cbg-treated PBMC. CFSE-labeled PBMCs stimulated with anti-3/28 mAbs were incubated with the substance for 72 h. A dose-dependent inhibition of cell proliferation was identified in all tested concentrations (3–100 μg/mL) (Figure 4A,B). At 3 μg/mL, an approximately 25% decrease in the proliferation of EC_50_ was determined at 14.8 μg/mL. Over 80% of inhibitory effects were investigated at 50 and 100 μg/mL.

### 2.6. Effects on the Functionality of PBMCs

**Figure 5:**
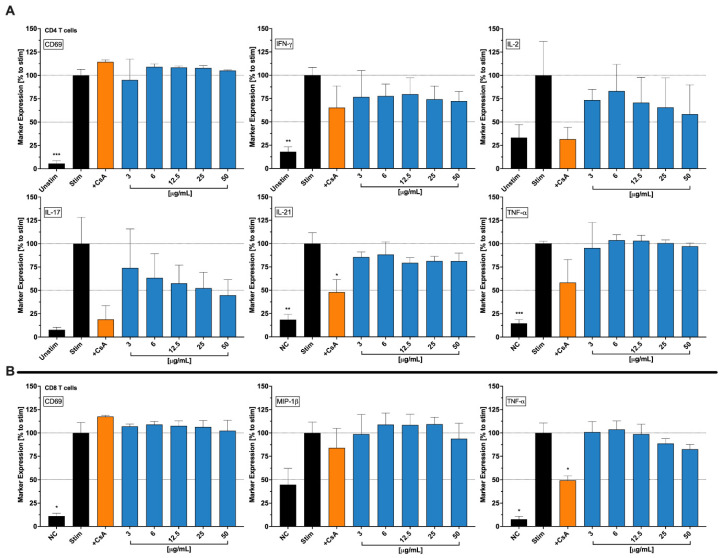
Impact of Cbg (blue) on the functionality of stimulated CD4^+^ T cells (**A**) and CD8^+^ T cells (**B**). After treatment with Cbg for 48 h, both T helper and cytotoxic T cells were explored separately for their functional activities using two flow cytometric staining panels. The activation markers and cytokines were normalized to untreated-stimulated cells and depicted as mean  ±  standard deviation. n  =  3; * *p*  <  0.05; ** *p* < 0.01; *** *p* < 0.001.

To assess the impact of Cbg on the surface expressions and secretion of cytokines, a multi-fluorescence panel has opted for the expression of CD69, IFN-γ, IL-2, IL-17, IL-21, and TNF-α for T helper cells (Figure 5A), and CD69, MIP1-β, and TNF-α for cytotoxic T cells (Figure 5B). Higher significance was obtained for cytokine and surface expressions in the anti-CD3/CD28-stimulated group compared to the negative control. The stimulated populations treated with different concentrations of Cbg were investigated for the inhibitory effects on pro-inflammatory-secreted cytokines/surface expressions.

Expression of the activation marker CD69 increased significantly in CD4^+^ helper T cells compared to the unstimulated negative control groups. For helper T cells, Cbg (3–50 μg/mL) suppressed IFN-γ with slightly less potent effects than the positive control CsA. Significant IFN-γ suppression of approximately 50% compared to the untreated stimulated control and was approximately 10% more potent than CsA. Cbg also decreased IL-2 and IL-17 in a concentration-dependent manner, with results starting as low as 3 μg/mL up to 100 μg/mL, with reductions of approximately 25% and 75%, respectively, compared to the untreated-stimulated group. In addition, IL-21 levels were reduced at all concentrations tested, with no substantial differences between concentrations, except at 100 μg/mL where a further reduction was observed. In addition, Cbg did not influence the expression of TNF-α cytokine secreted by the helper T cells except for 100 μg/mL, which showed a 20% reduction in the cytokine level.

As for CD8^+^ cytotoxic T cells, the CD69 marker expression also increased significantly in stimulated cells (Figure 5B). The decreased dose-dependent manner of TNF-α level was identified in Cbg-treated cytotoxic T cells starting from 25 μg/mL to 100 μg/mL. Although MIP-1β showed no effects when treated at low concentrations (3–25 μg/mL), a minor reduction at 50 μg/mL and an approximate reduction of 10% at 100 μg/mL were illustrated. Furthermore, the positive control CsA showed no strong effects, inhibiting the chemokine MIP-1β by only approximately 20%.

## 3. Discussion

This study examined anti-inflammatory impact of copper-bi-glycinate (CbG) on epithelial cells (HCE-T) and on primary immune cells (PBMCs), as well as on THP-1 and Jurkat cell lines. Copper is a natural element that is not only used in pipes and cable systems but is also an essential part of the human body that helps maintain homeostasis, including functional cell respiration and the immune system [10]. For example, superoxide dismutase (SOD) is one of the most important copper-dependent enzymes and is functionally associated with a wide variety of tissues and cells as an antioxidant [8]. 

Since the body uses this element, especially in the mitochondrial respiratory chain and antioxidant defense, we assumed it also enhances the HCE-T’ cells ability to downregulate reactive oxygen species ROS and, in that manner, aid wound healing abilities. Instead, we found no significant changes in any one of the previously described assays. Despite our findings, Ou et al. [31] described a slow-releasing hydrogel containing copper and selenium that mimics certain enzymes with the capacity to assist in the mitigation of ROS and the promotion of wound healing. We hypothesize that the lack of reduction of ROS, even though it is one of copper’s main tasks, might lie in the fact that the introduced form is not as similar to an enzyme of the human metabolism, as stated by Ou et al. [31], and to the particular toxicity at a higher dosage level.

In comparison, to these initial findings, we were able to show that the release process of the copper ion, when introduced into epithelial cells, plays a vital role for it’s toxic or healing effect, especially at high concentrations, as mentioned by Xiao et al. [32]. Our findings also underline the fact that copper in ionic form is toxic to cells if the necessary amount is exceeded (LC_50_ = 158 µg/mL). According to Nordberg et al. [33], the human body controls the uptake of copper via profuse expression of transmembrane proteins, including integral transporters such as copper-transporting ATPases 1 and 2. The toxicity of the ion itself stands in connection to its high reactivity and oxidative capability, which can lead to certain damages in human cells.

As previously discussed, copper plays a crucial role in human metabolism since it is a cofactor for many enzymes and is especially important in the mitochondrial cytochrome c oxidase., (and, therefore, the transport of electrons and the formation of adenosine triphosphate [ATP]). The importance of impeccable working mitochondria for wound healing is immense. Not only does impaired tissue need more energy to heal – hence the ATP – but it also does not need additional stress, which can increase the amount of ROS. This condition might worsen when there is no excellent antioxidant system, which is dependent on good-working mitochondria and the cofactor copper in the SOD [33]. From this point of view, using copper aid wound healing seems to be a good approach since it also shows antibacterial properties and induces epidermal growth factor in skin cells [32]. Numerous studies, have proven copper as a main actor in the wound healing of skin cells [34], especially regarding a common reason for poor healing conditions (bacterial infection), which respond well to metallic agents in, for example, nanoparticular form [34]. 

The necessity of copper for the organism, thus for the general cell itself, is clearly stated. However, it can also be toxic when dosed too high and/or introduced in an uncontrolled and abrupt way, as shown on tests conducted with brain cells [32]. Copper’s toxicity is associated with disturbances in the membrane potential of mitochondria, resulting in a lower ROS detoxification rate and subsequent to cell damage [35]. We did not establish our experimental setting with slow-releasing Cbg, which may be one reason for reduced wound healing at higher doses (50 µg/mL). Additionally, the inflicted wounds were not infected, and we can also assume that the cells were properly nourished so that the additional Cbg did not accelerate the healing process. Furthermore, the absorption of the monovalent ion is easier for the organism due to its high-affinity transporters for Cu (I) [35]. However, in our experimental setting, a Cu (II) ion was exposed to the cell lines, implying that the uptake of copper at lower doses may not be as effective as at higher (yet more toxic) one. Regarding these arguments, we saw no positive impact of Cgb on the wound healing process of HCE-T cells.

With regard to the proliferation of human PBMCs, we found an EC_50_ of 10.03 mg/mL for Cbg. A study by Singh and colleagues [24] also examined the influence of copper on the proliferation of PBMCs using a thymidine uptake assay, which showed a similar progressive reduction in cell proliferation. In 2016, Steinborn and colleagues [9] investigated the influence of copper nitrate on the proliferation of PBMCs and found a significant inhibition of proliferation at a concentration of 100 µM (≙18.8 µg/mL). Unfortunately, a comparison of the findings is only possible to a limited extent here, since Singh and colleagues used copper and Steinborn and colleagues use copper nitrate, while the results of this study are based on Cbg.

As for the surface and intracellular multifluorescence staining, our findings showed a progressive reduction of both interleukin (IL)-2 and interferon (IFN)-γ levels in CD4^+^ T cells, although at no significance. IL-17 levels, on the other hand, were strongly reduced in a progressive manner as the concentration increased, considering the relatively high standard deviation. Furthermore, Steinborn and colleagues also tested the secretion of IL-2 and IFN-γ for copper nitrate. For IL-2, the inhibition was significant at 100 µM (≙18.8 µg/mL), while for IFN-γ, there was a trend towards inhibition but no significance [9]. Moreover, Scuderi [23] showed that copper sulfate at a concentration of 0.25 mM inhibited IL-6 secretion, while concentrations above 0.03 mM inhibited IL-1b secretion and cell death was observed at concentrations above 1 mM. This study showed that in stimulated THP-1, when exposed to Cbg, the secreted levels of IL-6 and TNF-α were reduced. However, NF-κB expression was shown to be slightly increased. Zangiabadi and colleagues [22] showed that TNF-α, IFN-β, and IL-1β gene expressions, as well as NF-κB expression, were reduced in differentiated macrophage THP-1 cells when treated with copper-infused fabrics. The differences between these findings may be due to the previously mentioned fact that the beneficial properties of copper come with a well-proportioned dose and time, which can be realized through the copper-infused fabric, where the copper is certainly not forced out in an aggressive way. The expression of NF-κB, meanwhile, is connected over several pathways to the level of oxidative stress in the body, where it should help reduce TNF-α-induced ROS accumulation [36]. As mentioned earlier, a critical amount of copper can also induce ROS, leading to the hypothesis that the fast and high-dosage application of Cbg in our experimental findings, might have contributed to higher ROS levels and, therefore, increased NF-κB expression to reduce that ROS accumulation. IL-6 is known to assist the body in the first two stages of wound healing (proliferation and inflammation) [37], which makes us believe that, in our study, the reduction of IL-6 might be conclusive with the negative results from the wound healing assay with HCE-T cells. Reasons for this behaviour might be found in the previously mentioned points.

We further hypothesized that Cbg reduces the secretion of the cytokines and the proliferation through the binding of N-methyl-d-aspartate (NMDA) receptors. Copper is known to play an important role in regulating the excitability of neurons’ NMDA receptors [38]. Aside from the neurons, studies have shown that NMDA receptors’ protein and gene expressions are also detectable in human T cells, regulating their activation as well [39]. During the resting phase of T cells, NMDA ion channel receptors on the surface remain in a closed state. However, upon T cell activation, the membrane becomes depolarized and triggers NMDA receptors into an open state, which leads to an escalating level of intracellular Ca^2+^ [39,40,41]. Moreover, a study conducted by Orihara et al. showed that NMDA receptor agonists inhibited cytokine production, intracellular Ca^2+^ influx, and proliferation in primary Th1 CD4^+^ T cells. Thus, NMDA receptors have been suggested as one of the vital modulators of immune competence [42].

The Cbg is often used in food supplements [11], as it should have a high bioavailability [30], which to our best knowledge has not been investigated in detail. It is generally assumed that bioavailability increases when inorganic compounds, such as metal ions, are linked to stable organic compounds (similar to the body’s own structures) or consumed with phytates [43]. Unfortunately, the use of these more biologically, more identical structures is accompanied by a significant increase in the size of the molecule, which in turn might reduce stability and absorption rate. A fundamental difference between the mineral copper itself and bigger organic compounds is the chelation of the ion, which helps with absorption. Comparing copper sulfate with Cbg, there is one copper associated with one sulfate ion, whereas there are two bis-glycinates for one copper ion, which chelate the ion and help travel through organic barriers, all while hindering the copper ion from reacting early with other compounds. However, as shown by Shah [44], the inorganic copper compounds were also effective in treating anaemic rats. 

Various studies, particularly in relation to wound healing, have been conducted with copper sulfate, citrate, and oxide, but few have assessed Cbg, which is one of the reasons we chose to investigate this compound. As mentioned above, another reason is that this compound is common part of food supplements. However, this raises the question of whether Cbg’s *in vitro* use on a monolayer cell culture can have the same effect as an oral intake. It should also be noted that many studies concerning wound healing have used special galenic formulations (often as nanoparticles [34]), which particularly influence the duration and intensity of application and, thus, as already mentioned above, bring out the health-promoting aspects of copper more clearly. For supplementary reasons, copper compounds of sulfate, oxide, other amino acid chelates, and gluconates are consumed. Copper sulfate and other inorganic versions of copper, like copper oxychloride and hydroxide, have been used for a long time in agriculture to defend crops against fungi, algae, and molluscicides. They are utilized because micro-organisms are rather sensitive to their presence [45,46,47]. Despite the numerous positive properties of copper ions, other micronutrients such as iron, zinc, and selenium are primarily considered in the treatment of immune system disorders. It is possible that through use of copper in non-medical areas, (e.g., in agriculture) and it’s presumed consequences (e.g. increased copper content in soil and groundwater), immunological disorders are often not associated with copper deficiencies, as a higher intake is assumed.

Nevertheless, this micronutrient should continue to be the focus of research.

## 4. Material and Methods

### 4.1. Preparation of Copper Bis-Glycinate (Cbg)

Copper bis-glycinate (Cbg) was obtained from Sigma–Aldrich (Millipore Sigma, St. Louis, MO, USA). The Cbg stock was prepared by dissolving in dH_2_O to achieve a final concentration of 5 mg/mL. The solution was brought to a sonication bath at 40 °C for 10 min and subsequently vortexed.

### 4.2. Epithelial Cell Culture

The epithelial cell line is represented by SV40-immortalized human corneal epithelial transformed cells (HCE-T), which were acquired from the Riken Cell Bank (RCB2280). The cultivation and experiments were done according to the previous descriptions of Areesanan et al. [48]. The cultivation process used Dulbeccos-modified eagle medium F12 (DMEM/F-12 medium; Sigma–Aldrich (Millipore Sigma, St. Louis, MO, USA)) with the supplementation of 5% fetal calf serum (FCS: BioConcept, Allschwil, Basel, Switzerland), 5 µg/mL insulin, 10 ng/mL human epidermal growth factor (EGF), 0.5% DMSO, and 1% penicillin-streptomycin (all from Sigma–Aldrich (Millipore Sigma, St. Louis, MO, USA)). The cells were incubated at 37 °C and 5% CO_2_ and were passaged every 3–4 days. The following experiments with HCE-T were conducted in a 96-well plate with a seeding count of 2 × 10^4^ cells per well, except for the wound healing assay (described further below). The seeded cells were incubated overnight until they reached 90% confluence and washed twice, and the medium was replaced with a phenol red-, supplement-, and serum-free version for 24 h before the cells were introduced to Cbg.

### 4.3. Free-Radical Scavenging Activity

The protocol of Brand–Williams et al. served as a cell-free assay model to analyse free-radical scavenging activity [49]. A substance known as 2,2diphenyl-1-picrylhydrazyl (DPPH: Sigma–Aldrich (Millipore Sigma, St. Louis, MO, USA)) measures the potency of the substance to donate hydrogen to stabilize radical molecules of the DPPH, which causes the color to change from deep violet to clear color. A 1:2 serial dilution of Cbg (0.4–100 μg/mL) was mixed in 100% EtOH (Sigma–Aldrich, Millipore Sigma, St. Louis, MO, USA) with DPPH to achieve a final concentration of 100 nM of DPPH. The positive control was represented by Trolox, a water-soluble derivative of vitamin E that possesses excellent radical scavenging abilities. This was also diluted in EtOH (Sigma–Aldrich, Millipore Sigma, St. Louis, MO, USA) in the 1:2 serial way with a starting concentration of 100 µM. The DPPH-sample solutions were incubated at room temperature for 30 min in the dark. The absorptions were then measured spectrophotometrically at 517 nm (TECAN infinite M Plex; Tecan Group Ltd. Männedorf, Switzerland)). The following formula was used to calculate the percentage of the scavenging power compared to the blank, where the blank was pure EtOH (Sigma–Aldrich, Millipore Sigma, St. Louis, MO, USA) with DPPH without any samples.

$$DPPH\;scanvenging\;power\;\left[\%\right]=\frac{\left(blank-sample\right)}{blank}*100\%$$

### 4.4. Cell Viability for HCET Cells

Cytotoxicity assays were conducted to investigate the toxic concentrations of Cbg in HCE-T cells. Following the previously reported methodology [48], the cytotoxicity is shown when the reactant, water-soluble tetrazolin salt (WST-1: Sigma–Aldrich (Millipore Sigma, St. Louis, MO, USA)) is not reduced through the mitochondrial hydrogenase. If the cell is viable, there would be a color transition through the reduction process from red to yellow formazan. The cells were treated for 24 h at 37 °C and 5% CO_2_ with a serial dilution of Cbg (0.2–400 μg/mL). After washing the cells twice with phosphate-buffered saline (PBS: Sigma–Aldrich (Millipore Sigma, St. Louis, MO, USA)), they were exposed to a 1:10 *v*/*v* WST-1 assay solution. The absorbance scan was performed after incubation for 2 h at 37 °C and 5% CO_2_ at 450 nm with a spectrophotometer and a reference wavelength of 650 nm (TECAN infinite M Plex; Tecan Ltd. Group, Männedorf, Switzerland). Prior to the addition of WST-1 reagent, Triton-X-100 (0.01% *v*/*v* Sigma–Aldrich (Millipore Sigma, St. Louis, MO, USA)) was instituted as a positive control.

### 4.5. Intracellular ROS Measurement

For the execution of intracellular ROS measurement, 2′,7′-dichlorodihydrofluorescin diacetate (H_2_DCFDA: Sigma–Aldrich (Millipore Sigma, St. Louis, MO, USA)) was used. The utilization of membrane-permeable probes facilitates the quantification of reactive oxygen species (ROS) levels. These probes emit a fluorescent signal in response to ROS species, including hydroxyl and peroxyl radicals, as well as analogous active agents. As described by Areesanan et al. [48], the test was performed by incubating the HCE-T cells with 25 µm of H_2_DCFDA for 30 min at 37 °C. After washing the cells, they were treated with 0.4–100 μg/mL of Cbg for 10 min. After treatment, ROS level was induced by irradiating them with a wavelength of 312 nm, an intensity of 5.45 mW/cm^3^, and a dose of 50 mJ/cm^2^. Another incubation period of 30 min at 37 °C was implemented before the cells were measured at an excitation wavelength of 488 nm and an emission wavelength of 530 nm. Vitamin E derivative, Trolox (Tocris Bioscience, Bristol, UK), was used at a concentration of 100 µM as a positive control for reducing intracellular ROS levels. The measured results were presented as a percentage of control cells, which were irradiated but not treated.

### 4.6. Wound Healing Assay

To understand Cbg wound healing properties, an assay with scratched epithelial cells was executed. HCE-T cells were seeded in a 96-well plate at a concentration of 1.6 × 10^4^ per well and incubated for 24 h. The medium was changed to a depleted medium, which was a phenol-red non-supplemented medium containing 5% FCS to retard the wound healing after the “scratching” process. This adaption was necessary to ensure a monolayer and, therefore, enable the AutoScratch (BioTek, Agilent, Santa Clara, CA, USA) to create an analogue size of the scratches. The cells were scratched thrice at the same location to ensure cell-free gaps, washed twice with PBS, and afterwards treated with Cbg in a 1:10 serial dilution (0.05–50 µg/mL). Pure Hanks’ Balanced Salt solution (HBSS, Gibco (Fisher Scientific GmbH, Schwerte, Germany)) was applied as a positive control to hinder wound healing. The injured cells were incubated and photographed after 17 h with an inverted microscope (Zeiss, Carl Zeiss AG, Feldbach, Hombrechtikon, Switzerland). Quantitative results were obtained using ImageJ software (version: 2.14.0/1.54 g), where the percentage area of the existing wound was calculated.

### 4.7. THP-1, NF-κB-eGFP THP1 Reporter, and Jurkat Cell Culture

These cell lines were used to explore the effects of the Cbg on immune cells. Jurkat, a human T-lymphocyte cell line, was obtained from ATCC (Manassas, VA, USA), while immortalized human monocytic THP-1 and THP-1 NFκB cell lines were obtained from Merck (Darmstadt, Germany). For cell culture, a medium for non-adherent cells consisting of Roswell Park Memorial Institute Medium 1640 (RPMI-1640 medium; Sigma–Aldrich (Millipore Sigma, St. Louis, MO, USA) and the addition of 10% heat-inactivated FCS (BioConcept, Allschwil, Basel-Country), 2 mM L-glutamine, and 1% penicillin-streptomycin (both from Sigma–Aldrich (Millipore Sigma, St. Louis, MO, USA) was used. The cells were incubated at 37 °C and 5% CO_2_ and were passaged twice per week.

### 4.8. NF-κB Expression

This particular THP-1 NFκB cell line was coupled with an eGFP on the NF-κB-response element, which expresses an NF-κB response unit, which is marked with the fluorescent eGFP.

To test the impact of the Cbg on the NF-κB expression, the method was conducted as previously mentioned in Areesanan et al. [48]. The cells were inoculated in a 96-well plate (2 × 10^4^ cells/well) mixed with a 1:2 serial dilution of Cbg starting with 0.4 µg/mL to 100 µg/mL. To stimulate the production of NF-κB, the cells were exposed to 1 ng/mL lipopolysaccharide (LPS: Sigma–Aldrich, Millipore Sigma, St. Louis, MO, USA)). The untreated-stimulated cells serve as negative control. For a positive control group, dexamethasone (Dex: Sigma–Aldrich (Millipore Sigma, St. Louis, MO, USA)) was added in 0.1 µM to mitigate NF-κB expression. The batch was pretreated, which means the cells were exposed to Cbg or Dex for 30 min before further 24 h of incubation with the addition of LPS stimulation. To quantify the expressed signal, the cells were measured with a Beckmann Coulter flow cytometer, and data were analysed using FlowJo software (version: 10.10.0). The results are displayed as a percentage of the untreated-stimulated cells.

The viability was tested while they were sorted. Side scattering of living cells was more evident in comparison with dead and, therefore, not viable cells. The same cytometer and software, which were utilized in measuring the NF-κB expression, were used to gate the living cells. The results are shown in the percentage of stimulated cells.

### 4.9. Calcium Influx Assay

Jurkat cells, at a concentration of 5 × 10^6^, were resuspended in 1 mL of HBSS in an Eppendorf tube with a cocktail solution of 5 µM of Fluo3/AM (Sigma–Aldrich (Millipore Sigma, St. Louis, MO, USA)), 0.05% Pluronic Acid and 2 mM of Probenecid (both from Invitrogen, Waltham, MA, USA). The cells were incubated for 45 min at 37 °C, and the tube was inverted every 15 min before washing twice with HBSS. The cells (1 × 10^5^ cells/well) were then seeded in a 96-well plate and treated with different concentrations from 3 µg/mL to 100 µg/mL of Cbg and incubated at 37 °C for 10 min. Afterwards, the calcium baseline level was measured for 3 min with the 37 °C warm plate reader (TECAN infinite M Plex; Tecan Group Ltd. Männedorf, Switzerland). The cells were then stimulated using anti-CD3/aCD28 mAbs (1 µg/mL: Invitrogen, Waltham, MA, USA), and calcium level was measured again, this time for 10 min, on the plate reader. The results are presented as peak calcium levels in percentage to levels of untreated-stimulated cells after normalization to the levels of untreated-unstimulated cells after the following equation (X = measured fluorescent value):

$$Peak\;{Ca}^{2+}level=\frac{X-Unstim.}{Stim.-Unstim.}*100\%$$

### 4.10. THP1 Cell Viability and Cytokine Quantification

To investigate secreted IL-6, IL-8, and TNF-a cytokines, 5 × 10^4^ cells/well, except for the unstimulated control, were exposed to 1 µg/mL of LPS in a 96-well plate with 3–25 µg/mL of Cbg in culture medium for 6 h at 37 °C. For comparison, Dexamethasone (0.1µM: Sigma–Aldrich, Millipore Sigma, St. Louis, MO, USA) was set as a positive control for inhibiting the secretion of the early mediators. After treatment, the supernatants were collected and frozen at −80 °C until further analysis.

To better comprehend the following analysis of cytokines, the cell viability was also investigated using the WST-1 assay described earlier with the HCE-T cells. After collecting the supernatants, the cells were washed with PBS and incubated with 50 µL per well 10% *v*/*v* WST-1 in a phenol red-free medium for 2 h at 37 °C before they were measured spectrophotometrically at 450 nm. The results were calculated in percentage of the untreated-stimulated cells. Triton X-100 (1% *v*/*v*, Sigma–Aldrich, Millipore Sigma, St. Louis, MO, USA) was used as a positive control.

Furthermore, the expression of the secreted cytokines in the collected supernatants was observed using a multiplex bead-based flow cytometer assay (LEGENDplex, BioLegend, San Dieago, CA, USA) on a Beckmann Coulter (Brea, CA, USA) flow cytometer following the manufacturer’s protocol. The data were assessed via the FlowJo software and presented as a percentage of the stimulated cells.

### 4.11. Ethics Approval Statement

Every donor signed a written informed consent for blood collection. Each blood sample was drawn at the central blood donation of the University Hospital in Basel, Switzerland, in an anonymized and coded manner. In this experiment, three blood samples from different donors were used. No ID number of the samples is visible. Hence, the work does not fall under the scope of the Swiss Human Research Act, and no ethics approval from the Ethics Committee of Central and Northwestern Switzerland is required for this work with the blood samples obtained.

### 4.12. PBMC Isolation and Cell Culture

The human peripheral blood mononuclear cell (PBMC) represents a variety of immune active blood cells, such as monocytes, dendritic cells, and lymphocytes (e.g., T cells, B cells, and NK cells). The cultivation and preparation of PBMCs were performed following the protocol of Zimmermann–Klemd, A.M. et al. [50] and Winker et al. [51]. Briefly, the blood of donors was used to extract the PBMCs through density gradient medium centrifugation with Lymphorep™ (Stemcell Technologies, Vancouver, BC, Canada) in sugar gradient media (density: 1.077 g/cm^3^, 20 min, 500 g, 20 °C; Progen, Heidelberg, Germany). The buffy coat was extracted and washed with PBS before they were brought into RPMI 1640 medium supplemented with 10% FCS (GE Healthcare Life Sciences, Chicago, IL, USA), 2 mM of L-glutamine, and 100 µg/mL of penicillin–streptomycin (both from Sigma–Aldrich, Millipore Sigma, St. Louis, MO, USA). Finally, they were incubated at 37 °C and 5% CO_2_.

### 4.13. PBMC Multifluorescence Panel

As described in Winker et al. [51], after isolation, 5 × 10^5^ cells/well of a 96-well plate were stimulated with 100 ng/mL CD3/CD28 monoclonal antibodies (mAbs; eBioscience, San Diego, CA, USA), except for the unstimulated control cells, and treated with either Cbg in serial dilution (1:2) or positive control Cyclosporin-A (CsA, 5 µg/mL, Sandimmun, Novartis, Basel, Switzerland) for 48 h at 37 °C. Subsequently, using phorbol-12-myristat-13-acetate (PMA, 50 ng/mL; Sigma–Aldrich, Millipore Sigma, St. Louis, MO, USA) and ionomycin (1 µg/mL; Sigma–Aldrich, Millipore Sigma, St. Louis, MO, USA), the cells were restimulated except for the unstimulated ones. In addition, cytokine export was stopped at the golgi complex and at the endoplasmic reticulum (ER) by adding GolgiPlug™ (1 µL/mL; BDBiosiences, Franklin Lakes, NJ, USA) and GolgiStop™ (0.65 µL/mL; BDBiosciences, Franklin Lakes, NJ, USA), respectively. To ensure the effectiveness of the stimulation and blocking, the cells were incubated again for 4 h at 37 °C.

A different mixture for staining the surface proteins was prepared for two panels. The CD4 panel consisted of CD3/CD4, while the CD8 panel consisted of CD3/CD8 (Table 1), which were used to stain the cells for 30 min in the dark at RT. Afterwards, they were washed twice with fluorescent-activated cell sorting (FACS, consisting of PBS, 2% FCS and 0,1% Sodium azide (Millipore Sigma, St. Louis, MO, USA)) buffer and resuspended in Cytofix/Cytoperm (BD Biosiences, Franklin Lakes, NJ, USA) solution for 15 min at 4 °C. After washing twice with PermWash (BD Biosiences, Franklin Lakes, NJ, USA), the cells were then stained for 30 min at 4 °C with the targeted intracellular antibodies (Table 1). Afterwards, the cells were washed twice with PermWash and fixed for 10 min at 4 °C with 2% paraformaldehyde (PFA; Electron Microscopy Sciences, Hatfield, PA, USA). After resuspending the cells in FACS buffer, the measurement of fluorescent intensity was taken with a Beckmann Coulter FlowCytometer (Brea, CA, USA), along with the ensuing analysis with the FlowJo software (Version 10.10.0).

### 4.14. PBMC Proliferation Assay

The staining chemical carboxyfluorescein diacetate succinimidyl ester (CFSE, Millipore Sigma, St. Louis, MO, USA) was used to evaluate the influence of cell proliferation in the presence of Cbg. The cells were washed with PBS after isolation and brought to 5 × 10^6^ cells/mL. The staining preparation was conducted by exposing the cells to 5 µM of CFSE for 10 min at 37 °C. The cells were afterwards washed with the culture medium. Subsequently, the cells were stimulated with 100 ng/mL of CD3/CD28 mAbs and plated in a concentration of 2 × 10^5^ cells/mL with a volume of 100 µL per well. After a 72 h incubation period, they were analysed with a Beckmann Coulter FlowCytometer (Brea, CA, USA) and the FlowJo software (Version 10.10.0). Being able to inhibit cell proliferation CsA (5 µg/mL) was used as a positive control. The assay was conducted after the description of Winker et al. [51].

### 4.15. Statistical Analysis

All experiments were at least performed in independent triplicates. The values were expressed as mean ±  standard deviation. The analysis was conducted with the PRISM software (Graph Pad Software Version 10.4.1 (532) for PC). For the normality test, the Shapiro–Wilk test was used. Sample concentrations were tested for multiple comparisons using two-way Welch ANOVA and Dunnet’s T3 test. Values with * *p* < 0.05, ** *p* < 0.01, *** *p* < 0.001, and **** *p* < 0.0001 representing statistical significance.

## 5. Conclusions

The presented study demonstrated an immunomodulatory impact of copper on immune cells, which indicates a potentially positive influence on healing mechanisms of diseases with overwhelming immune system in the human body. Further research would be necessary to obtain that goal, but copper could be a very promising agent in inflammatory processes.

## Figures and Tables

**Table 1:** Surface and intracellular antibodies of panels 1 and 2 for multifluorescence staining.

	Surface Antibodies	
**Panel 1** (**helper T cells**)	**Panel 2** (**cytotoxic T cells**)	**Obtained from**
CD3-APC AlexaFluor750	CD3-APC AlexaFluor750	Beckman–Coulter
CD4-AlexaFluor700	CD8-AlexaFluor700	Beckman–Coulter
CD69-PC7	CD69-PC7	Beckman–Coulter
**Intracellular antibodies**		
**Panel 1** (**helper T cells**)	**Panel 2** (**cytotoxic T cells**)	**Obtained from**
IFN-γ FITC	-	Beckman–Coulter
TNF-α PE	TNF-α PE	Beckman–Coulter
-	MIP1-β BV421	Beckman–Coulter
IL-2 APC		BD
IL-17 PC5.5		BD
L-21 BV421		BD

## Data Availability

Data are contained within the article.

## Abbreviations

AR	Allergic rhinits
ATP	Adenosine triphosphate
BV	Bioviability
Cbg	Copper-bis-glycinate
CD 4/8/3/28	Cluster of differentiation
CFSE	Carboxyfluorescein diacetate succinimidyl ester
COX	Cytochrome oxidase
CsA	Cyclosporin-A
Dex	Dexamethasone
DMEM F12	Dulbeccos-modified eagle medium F12
DMSO	Dimethyl sulfoxide
DPPH	2,2 diphenyl-1-picrylhydrazyl
EC_50_	Effective concentration
EGF	Epidermal growth factor
ER	Endoplsamatic reticulum
FACS	Fluorescent-activated cell-sorting buffer
FCS	Fetal calf serum
H_2_DCFDA	2′,7′-dichlorodihydrofluorescin diacetate
HBSS	Hank’s balanced salt solution
HCE-T	Human corneal epithelial transformed cells
IFN*γ*	Interferon-gamma
Ig	Immunoglobulin
IL	Interleukin
IRF3	Interferon regulatory factor 3
LD_50_	Lethal dose
LOX	Lysine oxidase
LPS	Lipopolysaccharide
mAbs	Murine Antibodies
MIP1-β	Macrophage inflammatory protein 1-beta (Synonym: CCL4)
Nf-*κ*B	Nuclear factor ‘kappa-light-chain-enhancer’ of activated B-cells
NMDA	N-methyl-d-aspartate
PBMC	Human peripheral blood mononuclear cell
PBS	Phosphate buffered saline
PFA	Paraformaldehyd
PMA	Phorbol-12-myristat-13-acetate
ROS	Reactive oxygen species
RPMI 1640	Roswell Park Memorial Institute Medium
RT	Room temperature
SOD	Superoxide dismutase
Th2	Type 2 helper cells
THP1	Human leukemia monocyte cell line
TNF-α	Tumor necrosis factor alpha
WST-1	Water-soluble tetrazolin salt
